# Mapping and situation analysis of basic WASH facilities at households in Bangladesh: Evidence from a nationally representative survey

**DOI:** 10.1371/journal.pone.0259635

**Published:** 2021-11-04

**Authors:** Md. Sabbir Ahmed, Md Irteja Islam, Manik Chandra Das, Arifuzzaman Khan, Fakir Md Yunus

**Affiliations:** 1 Faculty of Nutrition and Food Science, Department of Community Health and Hygiene, Patuakhali Science and Technology University, Patuakhali, Bangladesh; 2 Faculty of Medicine and Health, School of Public Health, The University of Sydney, Camperdown, Sydney, NSW, Australia; 3 Centre for Health Research and School of Commerce, The University of Southern Queensland, Toowoomba, Queensland, Australia; 4 Maternal and Child Health Division, International Centre for Diarrhoeal Disease Research, Bangladesh (ICDDR, B), Mohakhali, Dhaka, Bangladesh; 5 School of Public Health and Life Sciences, University of South Asia, Dhaka, Bangladesh; 6 Asian Institute of Disability and Development, University of South Asia, Dhaka, Bangladesh; 7 School of Public Health, The University of Queensland, Herston, QLD, Australia; 8 Central Queensland Public Health Unit, Central Queensland Hospital and Health Service, Rockhampton, Australia; 9 Department of Psychology and Neuroscience, Dalhousie University, Halifax, Nova Scotia, Canada; University of Dhaka: Dhaka University, BANGLADESH

## Abstract

**Background:**

Ensuring water, sanitation, and hygiene (WASH) facilities for households remains a major public health concern in low- and middle-income countries (LMICs). This study investigated the current situation of basic WASH facilities for households in Bangladesh and drew a national coverage map.

**Methods:**

We analyzed the publicly available nationally representative 2019 Multiple Indicator Cluster Survey (MICS) dataset that was carried out by the Bangladesh Bureau of Statistics (BBS) with support from the United Nations Children’s Emergency Fund (UNICEF). A total of 61,209 households (weighted) were included in the analysis. Both bivariate and multivariate analyses were employed to examine the relationships between independent variables (socio-demographic and economic status) and their distributions over outcome variables (basic water, sanitation, and hygiene). Further, the spatial distribution of WASH facilities at the household level was depicted.

**Results:**

Coverage of access to basic water facilities at the household level was 99.5% (95% CI 99.4% to 99.6%), sanitation 60.7% (95% CI 60.0% to 61.5%), and hygiene 56.3% (95% CI 55.6% to 57.0%). However, coverage of combined access to all three components was 40.2% (95% CI 39.4% to 40.9%). Among all 64 administrative districts of Bangladesh, we found comparatively lower coverage of WASH facilities in the South and South-East regions and relatively higher in the households of the North and North-Western regions. An adjusted regression model revealed that richest households [AOR = 29.64, 95% CI 26.31 to 33.39], households in the rural areas [AOR = 1.64, 95% CI 1.50 to 1.79], household heads with higher educational attainment [AOR = 2.28, 95% CI 2.09 to 2.49], and households with 5+ family members [AOR = 1.64, 95% CI 1.56 to 1.71] had the higher likelihood to have basic WASH facilities.

**Conclusion:**

Less than half of the Bangladeshi households had access to all three major WASH components (basic water, sanitation, and hygiene facilities); however, variation exists at the individual parameter of basic water, sanitation, and hygiene facilities. A comprehensive WASH approach may reduce the gap and improve the quality of WASH facilities in Bangladesh.

## Introduction

Provisions of safe drinking water, sanitation, and hygiene (WASH) are vital for health and development, and is considered as one of the significant public health issues in the world [1,2]. Approximately 2.3 billion people still lack the basic sanitation and over half a billion lack the access to adequate water sources globally [3]. Evidence suggests that poor WASH conditions are directly linked with communicable diseases (e.g., diarrhea) and contribute significantly to the global disease burden [4,5]. It is estimated that about 88% of diarrheal disease is attributable to drinking contaminated water, improper sanitation, and poor hygiene. Furthermore, an earlier estimation suggested that nearly 2.4 million deaths (4.2% of all causes of deaths) worldwide could be avoided by adopting appropriate WASH practices [6,7]. To emphasize the magnitude of the existing WASH problem, United Nations (UN) included it as a standalone goal in the Sustainable Development Goal (SDG) (Goal#6 Ensure access to water and sanitation for all) [8].

In the 21^st^ century, WASH is considered as one of the main felt public health needs in low and middle-income countries (LMICs) [8]. Around half of its population has been suffering from one or more contaminated water and/or improper sanitation induced diseases or infections [9]. Despite Bangladesh being a densely populated LMIC country, it has achieved substantial improvement in basic WASH services such as improved drinking water, hand washing, eliminating open defecation; however, a significant proportion of the population is still exposed to unsafe WASH conditions [10,11]. Recent statistics indicated that just over one-third and half of the Bangladeshi population have access to safe drinking water and proper sanitation, respectively [12]. Previous 2012 Bangladesh Multiple Indicator Cluster Survey (MICS) reported that around 60% of the population followed proper handwashing techniques (i.e., with water and soap) at crucial times [13]. Regardless the low proportion, several recent studies reported that Bangladesh is on track of achieving SDG Goal #6 by 2030 emphasizing that schools and health facilities are reaching to a satisfactory hygiene level [11,14]. However, challenges still remain due to continued high population growth, rapid unplanned urbanization, infrastructure limitations, and experiencing frequent natural calamities such as events related to climate change and unusual flooding [10,14]. These challenges are directly linked with water contamination with fecal bacteria and heavy metals such as arsenic, manganese, and chloride [10,11]. Several WASH intervention trials that have been implementing in Bangladesh as well as other developing countries gave strong importance on the usefulness of an up-to-date mapping of the water and sanitation coverage [10,14,15]. Since there is no recent nationwide WASH coverage map in Bangladesh to the best of our knowledge, we aimed to reduce the knowledge gap by creating one using the latest nationally representative 2019 Bangladesh MICS dataset.

## Method

### Data source

We analyzed the latest publicly available nationally representative 2019 Bangladesh MICS dataset in this study [16]. This cross-sectional survey was a part of a sixth round of the global MICS initiative conducted by Bangladesh Bureau of Statistics (BBS) with support from the United Nations Children’s Emergency Fund (UNICEF). It captures various health indicators and household characteristics at the district level samples. This nationwide survey was carried out from January to June 2019.

### Sampling design and sample size

Data were collected at the household level and covered all administrative districts (N = 64) of Bangladesh using a two-stage stratified random sampling procedure. The urban-rural areas within each district were considered as the main sampling strata. Within each stratum, a specific number of census Enumeration Areas (EAs) were systematically selected with Probability Proportional to Size (PPS). After listing the households within the selected EAs, a systematic sample of 20 households were drawn from each Primary Sampling Unit (PSU). Total number of PSU and final sample size were 3,220 and 64,400, respectively. In this study, we used household dataset and the final sample size was 61,209 (weighted) after omitting the missing cases.

### Determination of WASH variables

We considered accessibility of basic water, sanitation, and hygiene facilities at households as the dependent variables of the study. These variables were further categorized according to the WHO-UNICEF Joint Monitoring Program (JMP) 2017 guidelines [17] as follows. ***Basic water*:** Drinking water from an improved source (i.e., piped water, borehole or tube wells, protected dug well, protected spring, rainwater, and packaged or delivered water), provided that the collection time is not more than 30 minutes for a round trip, including queuing. ***Basic sanitation*:** Use of improved sanitation facilities (i.e., flush/pour flush to the piped sewer system, septic tanks, or pit latrines; ventilated improved pit latrine, and composting toilets or pit latrines with slabs) that are not shared with other households. ***Basic hygiene*:** Availability of a handwashing facility having both soap and water on the household premises. Handwashing facilities may be fixed or mobile and may include a sink with tap water, bucket with taps, tippy-taps, and jugs or basins designated for handwashing. Soap includes bar soap, liquid soap, powder detergent, and soapy water. Lastly, we generated a new binary variable (Yes/No) for the households that combined all three basic facilities—referred as households having combined WASH facilities.

### Covariates

Socio-demographics and economic status (SES) of the households were considered as the independent variables for this study. These include (i) Division: eight administrative divisions of Bangladesh, (ii) Area: type of place of residence (rural or urban), (iii) Household wealth index: categorized based on the household assets and materials used to build house. The national wealth index quintile provided in the dataset was used for this wealth index variable. (iv) Educational status of household head: institutional educational level or grade attended, and (v) Family size: total family members currently living at the household (1–4 or 5+).

### Statistical analysis

We considered the sampling weight for the descriptive statistics as advised by the 2019 Bangladesh MICS dataset. Chi-square tests were performed to determine the association between the dependent and independent variables. Multivariate logistic regression models were constructed to estimate the adjusted odds ratio (AOR) at 95% confidence intervals. Those were found significantly associated in the bivariate analysis were included in the final regression models. All statistical tests were two-sided and considered significant at p < 0.05. Data were analyzed using Stata v14.2 (StataCorp, College Station, TX, USA). Considering the complex sample design of the survey we used Stata *‘svy’* command. We used Arc GIS v10.5 software for the spatial distribution of the coverage of WASH facilities across the 64 districts in Bangladesh.

### Ethical approval

Since the study followed secondary analysis of publicly available 2019 Bangladesh MICS dataset, ethical approval was not required. However, we obtained permission to analyze the dataset from the MICS team. Several research articles were published using this dataset in the similar manner. This MICS protocol was approved by the technical committee of the Government of Bangladesh lead by BBS. Participants gave their informed consent prior to the data collection. All respondents were informed about the confidentiality, anonymity and voluntary nature of participation. Additionally, respondents were informed of their right to refuse to answer all or any specific questions they felt uncomfortable with, as well as to withdraw from participation at any point of time. This MICS dataset is freely available on request at https://mics.unicef.org/surveys.

## Results

### Prevalence of access to basic WASH facilities

The prevalence of individual and combined WASH facilities by SES is presented in Table 1. Overall, 40.2% (95% CI 39.4% to 40.9%) of the households in Bangladesh had access to all three basic WASH facilities. At the disaggregated level, prevalence of having water, sanitation and hygiene facilities were 99.5% (95% CI 99.4% to 99.6%), 60.7% (95% CI 60.0% to 61.5%), and 56.3% (95% CI 55.6% to 57.0%), respectively. Coverage of individual and combined WASH facilities at households significantly varied across the eight administrative divisions in Bangladesh (p < 0.001). Prevalence of having combined WASH facilities was higher in the households of Rangpur division (48.3%), and lowest in Barishal division (25.2%). Urban areas had higher coverage of combined WASH facilities (51.8%) compare to the rural areas (36.9%). Households with the poorest wealth status had the lowest prevalence of combined WASH facilities (14.4%) whereas the richest had the highest (75.4%). Prevalence of combined WASH facilities at the households was positively associated with the higher educational level of the household head (p < 0.001), and increased number (+5) of family members (p < 0.001).

**Table 1:** Characteristics of the households and the prevalence of basic water, sanitation and hygiene facilities, (MICS 2019).

Variables		N (%)	Household having basic Water facility	Household having basic Sanitation facility	Household having basic Hygiene facility	Household having combined WASH facilities
			*% (95% CI)*	*% (95% CI)*	*% (95% CI)*	*% (95% CI)*
Division						
	Barishal	3479 (5.6)	99.1 (98.4–99.5)	64.1 (62.5–65.7)	34.1 (32.0–36.2)	25.2 (23.3–27.1)
	Chattogram	10733 (17.5)	99.4 (99.0–99.6)	64.4 (62.6–66.1)	49.8 (48.2–51.5)	37.5 (35.8–39.2)
	Dhaka	15502 (25.3)	100.0 (100.0–100.0)	56.4 (54.4–58.5)	56.9 (55.0–58.7)	40.9 (38.9–42.8)
	Khulna	7286 (11.9)	98.6 (98.1–99.0)	68.4 (67.1–69.7)	63.3 (61.8–64.8)	46.3 (44.8–47.8)
	Mymensingh	4560 (7.4)	99.8 (99.4–99.9)	53.5 (51.5–55.6)	47.3 (45.0–49.6)	30.2 (28.2–32.3)
	Rajshahi	8738 (14.2)	99.9 (99.5–100.0)	57.9 (56.3–59.6)	60.8 (59.0–62.6)	41.5 (39.8–43.2)
	Rangpur	7227 (11.8)	100.0 (100.0–100.0)	62.0 (60.4–63.5)	69.3 (67.8–70.8)	48.3 (46.7–49.8)
	Sylhet	3680 (6.0)	97.7 (96.0–98.7)	62.8 (59.9–65.6)	54.4 (51.5–57.3)	40.3 (37.6–43.0)
	***p value***		***< 0*.*001***	***< 0*.*001***	***< 0*.*001***	***< 0*.*001***
Area						
	Urban	13549 (22.1)	99.9 (99.8–100.0)	60.9 (58.7–63.1)	67.2 (65.1–69.2)	51.8 (49.5–54.0)
	Rural	47660 (77.9)	99.4 (99.2–99.5)	60.7 (60.0–61.4)	53.2 (52.5–53.9)	36.9 (36.2–37.6)
	***p value***		***< 0*.*001***	*0*.*818*	***< 0*.*001***	***< 0*.*001***
Wealth index						
	Poorest	12914 (21.1)	98.5 (98.1–98.9)	43.0 (41.8–44.1)	29.7 (28.7–30.8)	14.4 (13.6–15.2)
	Poorer	12445 (20.3)	99.7 (99.5–99.8)	54.4 (53.4–55.4)	46.9 (45.8–47.9)	27.0 (26.1–27.9)
	Middle	11889 (19.4)	99.7 (99.6–99.8)	64.1 (63.0–65.2)	56.5 (55.4–57.6)	38.3 (37.3–39.3)
	Richer	12009 (19.6)	99.7 (99.6–99.8)	64.2 (62.5–65.8)	65.1 (63.7–66.5)	48.3 (46.9–49.7)
	Richest	11950 (19.5)	99.9 (99.9–100.0)	79.7 (77.8–81.4)	85.7 (84.1–87.1)	75.4 (73.4–77.2)
	***p value***		***< 0*.*001***	***< 0*.*001***	***< 0*.*001***	***< 0*.*001***
Education of household head						
	Pre-primary or none	21431 (35.0)	99.4 (99.2–99.5)	53.1 (52.2–54.1)	45.5 (44.6–46.4)	28.9 (28.1–29.7)
	Primary	16585 (27.1)	99.4 (99.2–99.5)	55.9 (54.9–57.0)	50.9 (49.9–51.9)	33.1 (32.1–34.0)
	Secondary	15657 (25.6)	99.7 (99.5–99.7)	65.8 (64.8–66.9)	63.5 (62.4–64.6)	47.6 (46.5–48.6)
	Higher +	7536 (12.3)	99.8 (99.6–99.8)	82.2 (81.0–83.4)	83.9 (82.7–85.0)	72.4 (70.9–73.9)
	***p value***		***< 0*.*001***	***< 0*.*001***	***< 0*.*001***	***< 0*.*001***
Family member						
	1–4	37723 (61.6)	99.5 (99.4–99.6)	55.4 (54.5–56.3)	55.4 (54.6–56.3)	37.1 (36.3–37.9)
	5+	23486 (38.4)	99.4 (99.3–99.6)	69.3 (68.4–70.1)	57.6 (56.7–58.5)	45.1 (44.2–46.0)
	***p value***		***0*.*040***	***< 0*.*001***	***< 0*.*001***	***< 0*.*001***
**Overall**		**61209**	**99.5 (99.4–99.6)**	**60.7 (60.0–61.5)**	**56.3 (55.6–57.0)**	**40.2 (39.4–40.9)**

## District-level coverage of WASH facilities

**Fig 1:**
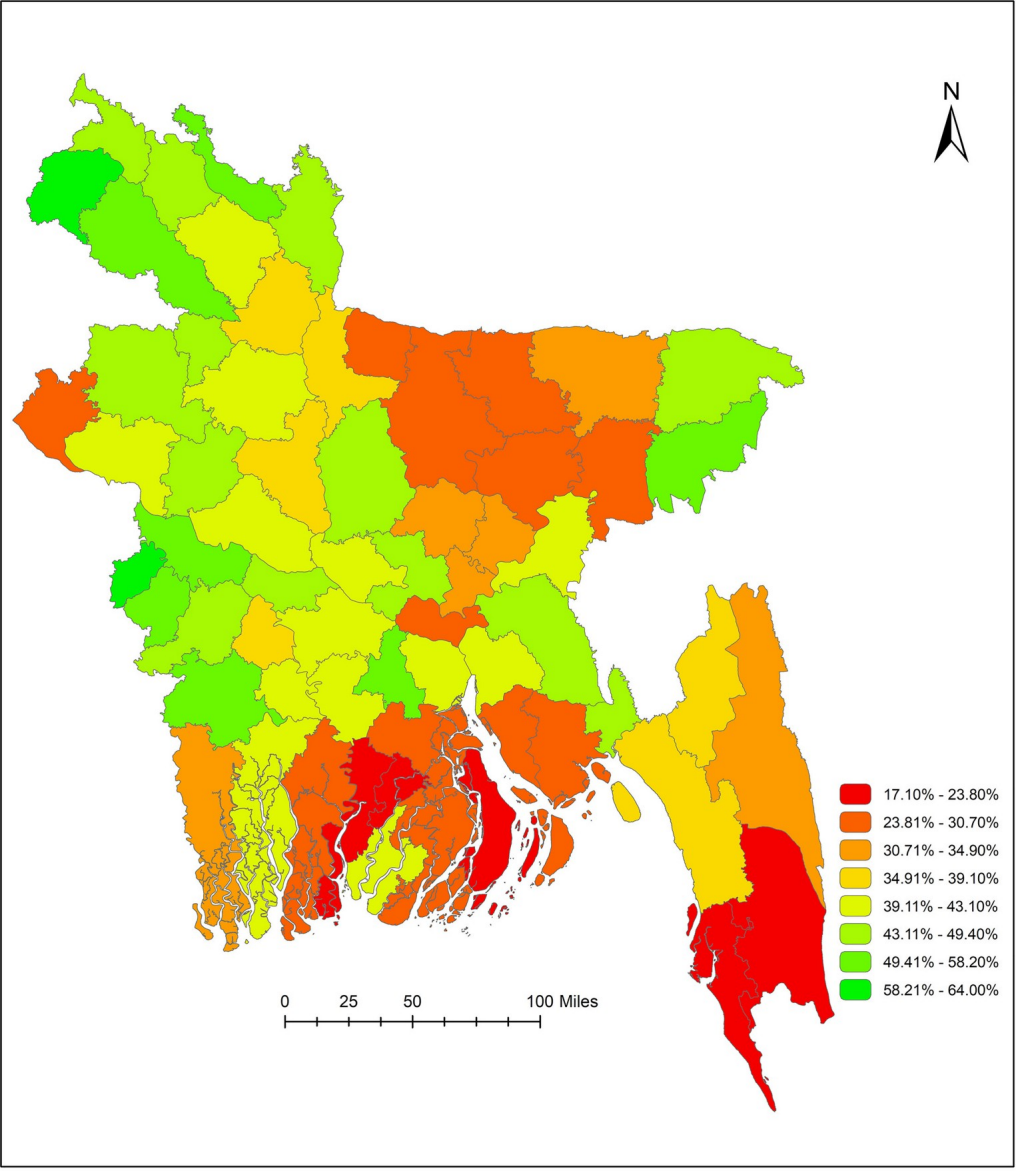
Coverage of basic WASH facilities (all three–water, sanitation, and hygiene facilities) in Bangladesh at the household level.

**Fig 2:**
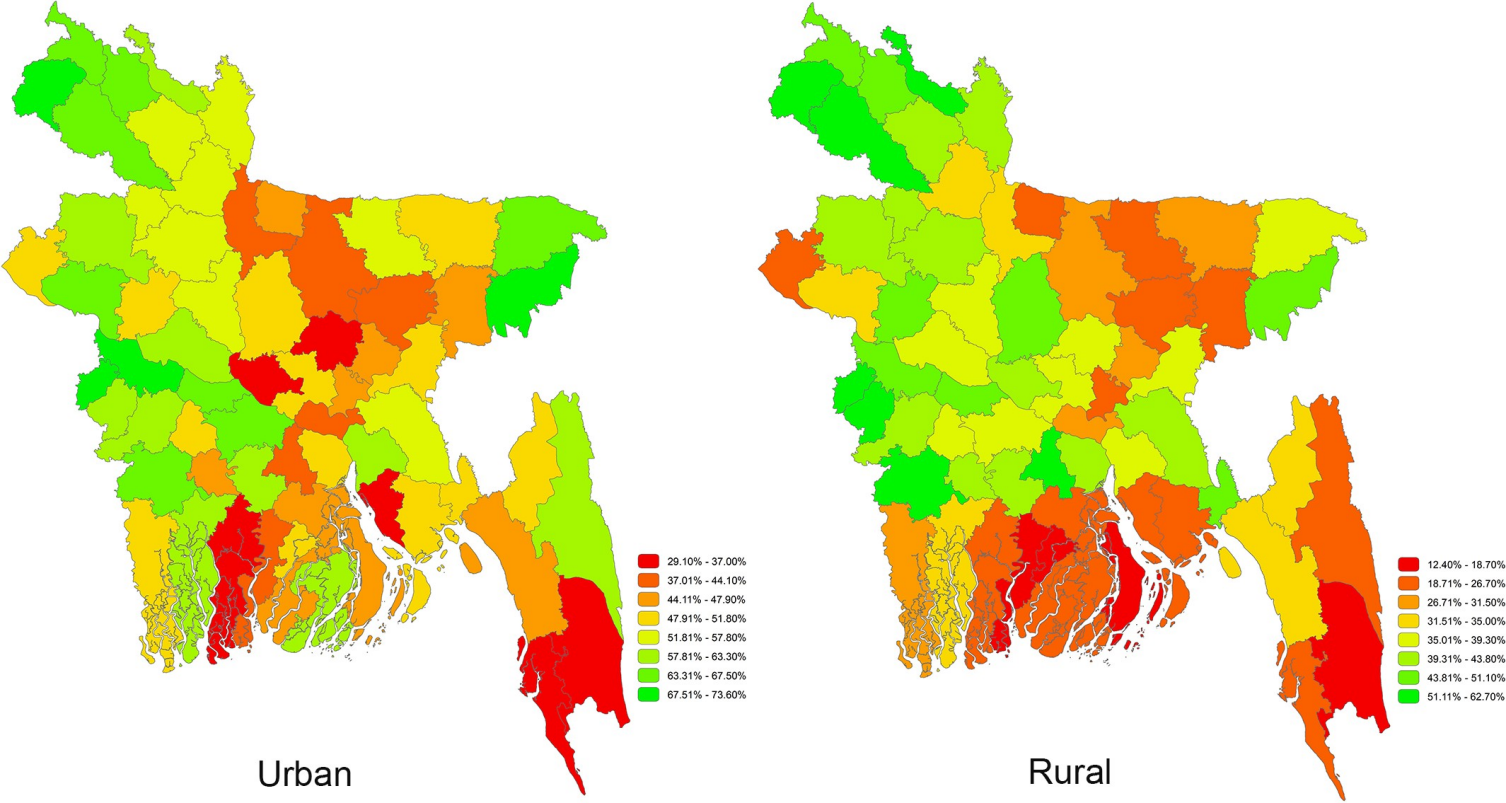
Coverage of basic WASH facilities by urban-rural areas in Bangladesh at the household level.

Fig 1 illustrates the district-level geographical distribution of combined WASH facilities across 64 districts of Bangladesh at the household level. Overall, South and South-East regions had comparatively lower, and North and North-western regions had relatively higher coverage of combined WASH facilities at the households. Of the 64 administrative districts, households in the Pirojpur district had the lowest (17.1%), and Meherpur district had the highest (64.0%) coverage of combined WASH facilities. Fig 2 illustrates that urban area in Bandarban district (29.1%) and rural areas in Pirojpur district (12.4%) had the lowest combined WASH coverage in Bangladesh. District-wise prevalence of combined WASH coverage is presented in S1 Table (S1 Table).

### Factors associated with WASH facilities

The association between SES and the accessibility of individual and combined WASH facilities is presented in Table 2. Administrative divisions, type of area, higher wealth index, higher educational status of the household head, and higher number of family members were identified as the independent predictors for the combined WASH facilities. The odds of having combined WASH facilities in Rangpur division were 3.5 times higher compared with Barishal division [AOR = 3.50, 95% CI 3.16 to 3.88] after adjusting the SES. In the adjusted model, rural areas had about 64% higher likelihood of having combined WASH facilities compared with urban areas [AOR = 1.64, 95% CI 1.50 to 1.79]; however, the odds for rural areas were lower compared to urban areas in the unadjusted model [OR = 0.54, 95% CI 0.49 to 0.59]—presented in S2 Table (S2 Table). Richest households had 29.6 times higher possibility of having combined WASH facilities at their households compared with the poorest households [AOR = 29.64, 95% CI 26.31 to 33.39]. Likelihood of having combined WASH facilities at households also increased with the higher educational attainment of the household head and with the higher number of family members compared to their counterparts, respectively.

**Table 2:** Factors associated with households having basic WASH facilities at household in Bangladesh.

Variables		Household having basic Water facility		Household having basic Sanitation facility		Household having basic Hygiene facility		Household having combined WASH facilities	
		AOR ^1^ (95% CI)	p value	AOR ^2^ (95% CI)	p value	AOR ^1^ (95% CI)	p value	AOR ^1^ (95% CI)	p value
Division									
	Barishal	Ref.		Ref.		Ref.		Ref.	
	Chattogram	0.81 (0.39–1.68)	0.582	0.64 (0.58–0.71)	< 0.001	1.06 (0.96–1.17)	0.230	0.84 (0.76–0.94)	0.002
	Dhaka	-		0.41 (0.37–0.46)	< 0.001	1.17 (1.05–1.31)	0.003	0.81 (0.72–0.92)	0.001
	Khulna	0.40 (0.20–0.78)	0.007	0.98 (0.88–1.07)	0.682	2.65 (2.39–2.93)	< 0.001	1.95 (1.75–2.16)	< 0.001
	Mymensingh	4.74 (1.28–17.55)	0.020	0.60 (0.54–0.67)	< 0.001	1.82 (1.61–2.06)	< 0.001	1.28 (1.12–1.46)	< 0.001
	Rajshahi	4.81 (1.18–19.52)	0.028	0.68 (0.62–0.75)	< 0.001	2.86 (2.58–3.17)	< 0.001	1.94 (1.74–2.16)	< 0.001
	Rangpur	-		0.91 (0.83–1.00)	0.078	5.37 (4.83–5.97)	< 0.001	3.50 (3.16–3.88)	< 0.001
	Sylhet	0.29 (0.13–0.63)	0.002	0.66 (0.58–0.76)	< 0.001	1.66 (1.44–1.92)	< 0.001	1.21 (1.06–1.38)	0.004
Area									
	Urban	Ref.		Ref.		Ref.		Ref.	
	Rural	0.30 (0.14–0.62)	0.001	2.07 (1.88–2.29)	< 0.001	1.43 (1.31–1.56)	< 0.001	1.64 (1.50–1.79)	< 0.001
Wealth index									
	Poorest	Ref.		Ref.		Ref.		Ref.	
	Poorer	3.80 (2.48–5.84)	< 0.001	1.59 (1.49–1.69)	< 0.001	1.99 (1.87–2.13)	< 0.001	2.11 (1.95–2.28)	< 0.001
	Middle	5.13 (3.23–8.14)	< 0.001	2.36 (2.19–2.53)	< 0.001	3.36 (3.12–3.62)	< 0.001	4.00 (3.67–4.34)	< 0.001
	Richer	5.00 (2.76–9.05)	< 0.001	2.43 (2.24–2.65)	< 0.001	5.43 (5.00–5.89)	< 0.001	6.92 (6.32–7.58)	< 0.001
	Richest	16.55 (7.68–35.64)	< 0.001	5.27 (4.67–5.95)	< 0.001	20.39 (17.92–23.20)	< 0.001	29.64 (26.31–33.39)	< 0.001
Education of household head									
	Pre-primary or none	Ref.		Ref.		Ref.		Ref.	
	Primary	0.91 (0.70–1.17)	0.474	0.97 (0.92–1.02)	0.272	1.00 (0.95–1.06)	0.778	0.93 (0.88–0.98)	0.018
	Secondary	1.06 (0.79–1.43)	0.675	1.23 (1.16–1.30)	< 0.001	1.23 (1.16–1.31)	< 0.001	1.22 (1.15–1.30)	< 0.001
	Higher +	0.75 (0.48–1.18)	0.224	2.29 (2.08–2.52)	< 0.001	2.36 (2.15–2.60)	< 0.001	2.28 (2.09–2.49)	< 0.001
Family member									
	1–4	Ref.		Ref.		Ref.		Ref.	
	5+	1.01 (0.81–1.25)	0.917	1.93 (1.85–2.02)	< 0.001	1.23 (1.17–1.28)	< 0.001	1.64 (1.56–1.71)	< 0.001

AOR = Adjusted Odd Ratio.

^1^ Adjusted with division, area, wealth index, education of household head and family member.

^2^ Adjusted with division, wealth index, education of household head and family member.

## Discussion

We used the latest country representative publicly available 2019 Bangladesh MICS dataset to capture the situation of WASH facilities in Bangladesh at the household level. We found a wide range of variation of having the combined WASH facilities (17.1% to 64.0%) across the 64 districts of Bangladesh. Large variations were also observed among the individual WASH components. For instance, prevalence of basic water facilities at the household level was 99.5%, whereas the prevalence of hygiene facilities was 56.3%. Although Bangladesh has made substantial progress in reducing the open defecation from 34% in 1990 to 1.5% in 2019, the quality of the sanitation facilities remained a major concern; about 20% of sanitation facilities are classified as ‘limited service’, and around 14% as ‘unimproved’ [16,18]. We found the overall coverage of combined WASH facilities was 40.2%. Comparing to the earlier 2012 Bangladesh MICS data, our analysis revealed that sanitation facilities have slightly improved (55.9% to 60.7%), and hygiene facilities have marginally decreased (59.1% to 56.3%) over the 6–7 years (MICS 2012–13 to MICS 2019) [13]. It indicates the inconsistent progression across the individual component of WASH facilities in Bangladesh. As a result, Bangladesh may lose its success on the progression of the WASH indicators and may not be able to reach the SDGs if a comprehensive WASH approach is not adopted.

Prevalence of having combined WASH facilities at the households significantly varied across the eight administrative divisions and 64 districts of Bangladesh. Combined WASH coverage was the highest (48.3%) in Rangpur, northern division of Bangladesh which consists of 8 districts and lowest (25.2%) in Barishal division, a south-central part of the country with 6 districts. Although we found that urban areas (51.6%) had a higher prevalence of combined WASH facilities compared to the rural areas (36.9%), the likelihood of having the combined WASH facilities at the household was 1.6 times higher in the rural areas compared to the urban areas after adjusting geographical region, household wealth index, education attainment of the household head and the number of family members in the household. While the urban areas offer better life amenities compared to the rural areas, the reasons behind the higher odds in rural areas are not entirely clear. Inclusion of densely populated slums in the urban areas may have contributed to this shift. Earlier studies in Bangladesh supported our findings that poor WASH facilities exist in urban slums [19]. However, an opposite finding was also reported in a study carried out in India that found significant disparity in the coverage of WASH facilities between rural (lower coverage) and urban areas, which is similar to our study findings [20]. Nevertheless, future cluster studies might explain such facts.

Our results suggest that the higher the wealth of the households, the higher the coverage of combined WASH facilities. An earlier political-economy analysis study carried out in Cambodia, Ghana and Nepal supported such relationship [21]. Furthermore, WHO-UNICEF JMP indicated a similar relationship that the access to WASH is significantly higher in the second poorest quintile compared to the poorest quintile countries [17]. Lack of the combined WASH facilities expose the poorer households to a higher vulnerability towards the water-borne diseases. It is widely evident and acknowledged that poor coverage of WASH is one of the major drivers for the higher burden of infectious diseases in the LMICs [22]. Previous research endorsed the potentiality of promotional approaches to increase the utilization of combined interventions in the LMICs [23]. However, it is crucial to determine the existing coverage of basic WASH facilities and its associated factors at the household levels beforehand of such promotional approaches. A recent geostatistical modeling reported that inequities exist in accessing to drinking water and sanitation facilities in the LMICs during the period of 2000–2017 [24]. Our study identified the disparities in the coverage of the WASH facilities by district across Bangladesh and presented in a pictorial format for the policymakers to have the knowledge on the most recent situation in Bangladesh. The coverage map and associated factors generated from our study could be an essential precursor for the future WASH experimental trials and for designing an informed policy-oriented public health program incorporating the effective interventions for the prevention of the WASH related morbidities in Bangladesh.

Educational status of the household head is another factor that was associated with the coverage of combined WASH facilities in Bangladesh. We found that the higher the education level of the household head, the higher the combined WASH coverage. It is likely because higher education of the household head had the knowledge of the health benefits of having WASH facilities in their respective households. Furthermore, household wealth may have also allowed for access to have more education in addition of being able to cover the required costs of the WASH facilities in their households. Moreover, several studies found significant positive relationship between higher educational attainment and improved WASH [25–27]. Although it may not be possible to improve the institutional form of education of the household heads at the later stage of their life, large-scale public health WASH promotional campaigns focusing on the access to hygiene facilities may improve the overall WASH situation in Bangladesh. School-based WASH approach could be another promising way to improve such situation. Several earlier studies focused on the school-based WASH approach and found overall positive result in improving WASH practices in the households [28–31]. We also observed a positive association of increased numbers of family members with the availability of individual and combined WASH components at households. In our previous study we found that smaller family size (1–4) had higher odds of not practicing handwashing in Bangladesh [32], which is in line with the findings of this study. Based on our experience living in Bangladesh this association can be explained by the fact that larger families necessitated more and frequent use of WASH facilities and difficult and may not have the opportunity to share with other families. Regardless of their economic status, larger families may spend more to ensure basic WASH facilities at households.

We acknowledge several limitations of our study beyond the strength of a large-scale nationally representative 2019 Bangladesh MICS survey. Although this survey adopted a rigorous approach to data‐quality assurance and can explain the generalizability of the findings, but because of the cross-sectional nature of the study design our findings failed to establish causal inferences. Caution is advised while interpreting the results. Self-reported WASH facility data is another limitation of the study since it may be a subject to reporting bias and may have overestimated actual usage [33]. Regardless, the study design is strong enough to capture the current WASH situation and its associated SES factors in Bangladesh.

## Conclusion

Overall, less than half of the households in Bangladesh have all three major components of WASH facilities. Households that are wealthy and are in the rural areas, have the higher educational attainment of household head and households comprising of 5+ family members contributed to the higher access to combined WASH facilities. At the disaggregated level, the study concludes satisfactory coverage of basic water facilities across Bangladeshi households; however, significant progressions are to be made on the sanitation and hygiene elements of WASH to achieve the SDG #6. The study findings shed light on the importance of collective efforts from different sectors (e.g., Government, Non-Government organizations, and private sectors) in improving the WASH components in Bangladeshi households. Furthermore, the results of the study offer an opportunity for the researcher, public health programs professionals and policymakers to design a comprehensive WASH intervention package to areas in Bangladesh where it warrants and to develop plans for a sustainable WASH solution for Bangladesh as well as to set an example for the LMICs.

## Supporting information

S1 Table District wise distribution of the prevalence of the households with basic WASH facilities, MICS-2019. (PDF)

S2 Table Logistic regression analysis (crude) between the basic WASH facilities and study variables. (PDF)
