# Supplementary material for: Mapping and situation analysis of basic WASH facilities at households in Bangladesh: Evidence from a nationally representative survey
Source: PLoS One. 2021 Nov 4;16(11):e0259635. doi: 10.1371/journal.pone.0259635 (PMC8568162; doi:10.1371/journal.pone.0259635)
Supplement: S1 Table — (PDF) [file pone.0259635.s001.pdf]

Supplementary Table 1. District wise distribution of the prevalence of the households with basic WASH facilities, MICS-2019.

Sl no.	Division	District	Overall (%)	Urban (%)	Rural (%)
1	Barishal	Barishal	29.8	46.8	25.0
2		Bhola	21.6	45.9	18.0
3		Barguna	43.1	47.9	21.9
4		Jhalakathi	23.8	50.0	18.7
5		Patuakhali	28.9	62.8	24.4
6		Pirojpur	17.1	43.6	12.4
7	Chittagong	Bandarban	17.2	29.1	13.3
8		Brahmanbaria	40.8	51.8	38.7
9		Chandpur	41.8	62.5	37.0
10		Chittagong	38.8	46.0	34.2
11		Cumilla	45.1	57.8	42.8
12		Cox's Bazar	23.7	36.0	20.1
13		Feni	46.6	51.8	45.3
14		Khagrachari	37.9	50.0	33.0
15		Lakshimipur	27.9	37.0	26.3
16		Noakhali	29.0	50.3	25.1
17		Rangamati	33.8	60.2	24.8
18	Dhaka	Dhaka	46.7	50.1	36.6
19		Faridpur	42.3	66.4	38.3
20		Gazipur	34.9	31.4	36.3
21		Gopalgonj	42.5	60.4	40.6
22		Kishoreganj	28.4	41.8	26.1
23		Madaripur	53.5	44.1	54.9
24		Manikgonj	40.2	35.4	40.5
25		Munshigonj	30.6	41.8	29.1
26		Narayangonj	32.6	45.8	26.1
27		Narshindi	33.7	46.0	30.4
28		Rajbari	48.8	64.9	46.6
29		Shariatpur	41.9	50.9	40.8
30		Tangail	45.8	50.0	45.1
31	Mymensingh	Mymensingh	30.7	41.8	28.7
32		Jamalpur	36.0	43.0	34.6
33		Sherpur	26.6	47.4	23.6
34		Netrokona	25.3	55.9	21.5
35	Khulna	Bagerhat	28.2	37.0	26.7
36		Chouadanga	56.0	63.3	53.3
37		Jessore	58.2	67.3	56.4
38		Jhenaidah	44.9	62.2	41.9
39		Khulna	41.8	62.0	33.2
40		Kustia	53.7	71.7	51.1
41		Magura	38.3	49.1	36.7
42		Meherpur	64.0	70.7	62.7
43		Narail	41.6	44.9	40.8
44		Shatkhira	33.4	48.9	31.5

45	Rajshahi	Bogra	43.1	53.9	40.5
46		Jaipurhat	44.8	57.7	42.4
47		Naogaon	45.6	61.7	43.8
48		Natore	46.0	50.5	45.4
49		Chapai Nawabganj	27.3	51.4	21.5
50		Pabna	40.5	59.4	37.1
51		Rajshahi	42.6	64.9	33.4
52		Sirajganj	39.1	54.2	36.8
53	Rangpur	Dinajpur	57.5	66.2	56.0
54		Gaibandha	36.6	54.4	35.0
55		Kurigram	44.6	53.8	42.9
56		Lalmonirhat	56.1	60.8	55.7
57		Nilphamari	48.2	64.5	45.5
58		Panchagrah	49.4	67.5	47.5
59		Rangpur	42.1	55.4	40.1
60		Thakurgaon	60.5	68.9	59.5
61	Sylhet	Hobiganj	28.4	44.9	26.2
62		Moulovibazar	52.1	73.6	49.6
63		Sunamgonj	32.5	51.1	30.5
64		Sylhet	46.7	66.5	39.3

MICS = Multiple Indicator Cluster Survey
