# Supplementary material for: Mapping and situation analysis of basic WASH facilities at households in Bangladesh: Evidence from a nationally representative survey
Source: PLoS One. 2021 Nov 4;16(11):e0259635. doi: 10.1371/journal.pone.0259635 (PMC8568162; doi:10.1371/journal.pone.0259635)
Supplement: S2 Table — (PDF) [file pone.0259635.s002.pdf]

Supplement Table 2. Logistic regression analysis (crude) between the basic WASH facilities and study variables.

Variables	Household having basic Water facility		Household having basic Sanitation facility		Household having basic Hygiene facility		Household having all three basic facilities	
	COR (95% CI)	*p value*	COR (95% CI)	*p value*	COR (95% CI)	*p value*	COR (95% CI)	*p value*
Division								
Barishal	Ref.		Ref.		Ref.		Ref.	
Chattogram	1.46 (0.73 – 2.91)	0.278	1.01 (0.91 – 1.12)	0.810	1.92 (1.71 – 2.15)	< 0.001	1.78 (1.57 – 2.01)	< 0.001
Dhaka	1		0.72 (0.65 – 0.81)	< 0.001	2.55 (2.26 – 2.87)	< 0.001	2.05 (1.80 – 2.33)	< 0.001
Khulna	0.66 (0.35 – 1.25)	0.209	1.21 (1.10 – 1.33)	< 0.001	3.34 (2.98 – 3.74)	< 0.001	2.56 (2.28 – 2.88)	< 0.001
Mymensingh	5.39 (1.45 – 19.96)	0.012	0.64 (0.57 – 0.72)	< 0.001	1.73 (1.52 – 1.98)	< 0.001	1.28 (1.11 – 1.48)	< 0.001
Rajshahi	6.76 (1.69 – 26.94)	0.007	0.77 (0.70 – 0.85)	< 0.001	3.00 (2.66 – 3.39)	< 0.001	2.10 (1.86 – 2.38)	< 0.001
Rangpur	1		0.91 (0.82 – 1.00)	0.063	4.37 (3.88 – 4.92)	< 0.001	2.77 (2.46 – 3.12)	< 0.001
Sylhet	0.40 (0.18 – 0.90)	0.027	0.94 (0.82 – 1.08)	0.439	2.30 (1.98 – 2.68)	< 0.001	2.01 (1.72 – 2.33)	< 0.001
Area								
Urban	Ref.		Ref.		Ref.		Ref.	
Rural	0.12 (0.06 – 0.25)	< 0.001	0.98 (0.89 – 1.08)	0.818	0.55 (0.50 – 0.61)	< 0.001	0.54 (0.49 – 0.59)	< 0.001
Wealth index								
Poorest	Ref.		Ref.		Ref.		Ref.	
Poorer	4.33 (2.90 – 6.47)	< 0.001	1.58 (1.49 – 1.68)	< 0.001	2.08 (1.95 – 2.21)	< 0.001	2.20 (2.04 – 2.38)	< 0.001
Middle	5.11 (3.33 – 7.86)	< 0.001	2.36 (2.21 – 2.52)	< 0.001	3.07 (2.87 – 3.28)	< 0.001	3.70 (3.42 – 4.00)	< 0.001
Richer	5.49 (3.26 – 9.22)	< 0.001	2.37 (2.18 – 2.59)	< 0.001	4.41 (4.07 – 4.77)	< 0.001	5.57 (5.11 – 6.07)	< 0.001
Richest	24.04 (12.01 – 48.08)	< 0.001	5.20 (4.60 – 5.87)	< 0.001	14.10 (12.36 – 16.08)	< 0.001	18.22 (16.17 – 20.52)	< 0.001
Education of household head								
Pre-primary or none	Ref.		Ref.		Ref.		Ref.	
Primary	0.98 (0.76 – 1.26)	0.909	1.11 (1.06 – 1.17)	< 0.001	1.24 (1.18 – 1.30)	< 0.001	1.21 (1.15 – 1.27)	< 0.011
Secondary	1.79 (1.34 – 2.39)	< 0.001	1.70 (1.61 – 1.79)	< 0.001	2.08 (1.97 – 2.19)	< 0.001	2.23 (2.12 – 2.35)	< 0.001
Higher +	2.56 (1.60 – 4.08)	< 0.001	4.07 (3.74 – 4.44)	< 0.001	6.23 (5.70 – 6.80)	< 0.001	6.47 (5.97 – 7.01)	< 0.001
Family member								
1-4	Ref.		Ref.		Ref.		Ref.	
5+	0.78 (0.62 – 0.98)	0.041	1.81 (1.74 – 1.89)	< 0.001	1.09 (1.04 – 1.13)	< 0.001	1.39 (1.33 – 1.44)	< 0.001

COR = Crude Odd Ratio; Ref. = Reference category
